# Polyploidization of *Indotyphlops braminus*: evidence from isoform-sequencing

**DOI:** 10.1186/s12863-024-01208-y

**Published:** 2024-02-26

**Authors:** Fei Zhu, Jing Lu, Ke Sun, Cao Deng, Yu Xu

**Affiliations:** 1 School of Life Sciences, Guizhou Normal University, 550025 Guiyang, Guizhou China; 2 Department of Bioinformatics, DNA Stories Bioinformatics Center, 610000 Chengdu, China

**Keywords:** *Indotyphlops braminus*, Polyploidization, Transcriptome, Sequencing, Evolution

## Abstract

**Background:**

*Indotyphlops braminus*, the only known triploid parthenogenetic snake, is a compelling species for revealing the mechanism of polyploid emergence in vertebrates.

**Methods:**

In this study, we applied PacBio isoform sequencing technology to generate the first full-length transcriptome of *I. braminus*, aiming to improve the understanding of the molecular characteristics of this species.

**Results:**

A total of 51,849 nonredundant full-length transcript assemblies (with an N50 length of 2980 bp) from *I. braminus* were generated and fully annotated using various gene function databases. Our analysis provides preliminary evidence supporting a recent genome duplication event in *I. braminus*. Phylogenetic analysis indicated that the divergence of *I. braminus* subgenomes occurred approximately 11.5 ~ 15 million years ago (Mya). The full-length transcript resource generated as part of this research will facilitate transcriptome analysis and genomic evolution studies in the future.

**Supplementary Information:**

The online version contains supplementary material available at 10.1186/s12863-024-01208-y.

## Background

*Indotyphlops braminus*, previously known as *Ramphotyphlops braminus* [1–3], is classified in the genus *Indotyphlops* of the family Typhlopidae. It is one of the smallest snake species, with a body length of 7–17 cm. Although its origin was speculated to be southern or eastern Asia, *I. braminus* is now identified as a globally invasive species. It has been documented in tropical and subtropical regions worldwide, except South America [1, 4, 5]. The species is predominantly located in provinces south of the Yangtze River in China [6]. The extensive invasion of *I. braminus* can be partly attributed to the potted plant trade and its ability for parthenogenetic reproduction [3, 7]. Parthenogenesis, a reproductive strategy where a female can reproduce without male involvement to create an entire population [8, 9], has been widely studied for its long-term consequences [10, 11]. As the only truly parthenogenetic vertebrates, reptiles provide critical insights into the persistence of sexual reproduction [11, 12]. Furthermore, *I. braminus* is an allotriploid species, that results from hybridization [2, 3, 5]. Allopolyploids potentially benefit from heterosis, by harbouring multiple gene copies that can evolve new or varied functions, facilitating niche expansion and adaptation to environmental changes [13]. McDowell’s [14] initial proposal that *I. braminus* is an all-female species was later confirmed by Nussbaum [1]. Nevertheless, limited information is available regarding the reproductive characteristics of this diminutive snake [7]. Wynn et al. [2] and Ota et al. [3] demonstrated that *I. braminus* as a triploid asexual species according to karyotyping. While karyotyping offers initial evidence, additional molecular biology research is necessary. Elucidating the timing and mechanism of polyploidy in this snake species will yield insights crucial for future research into polyploid vertebrates. A recent publication presented *I. braminus* draft genome, which exhibited a total length of 1.86 Gbp and an N50 scaffold size of 1.25 Mbp, indicative of a potentially chimeric single haplotype [5]. Acquiring high-quality genomic or transcriptomic data is vital for advancing related research.

Full-length transcriptome data enhance the understanding of gene content and refine genome annotation, facilitating detailed analysis of gene structure and transcriptional information [15, 16]. Polyploids, which have multiple chromosome sets, typically exhibit more intricate transcriptomes than diploids [17, 18]. In polyploids, duplicated genes may result in redundancies, potentially introducing new functions [19]. Additionally, a link might exist between an increase in gene numbers or genomes and phenotype complexity [20]. Assembling transcripts from complex polyploid genomes accurately can be challenging when using short-read sequencing technologies. Short-read sequencing risks errors, such as merging similar gene copies into one contig [21]. Single-molecule long-read sequencing, represented by isoform sequencing (Iso-seq) by Pacific Biosciences (PacBio), excels in accurately analysing transcript structural information [22, 23]. This approach is particularly advantageous for polyploid species due to its ability to differentiate homeologs [24, 25]. Long-read sequencing, which covers the entire transcript, can resolve complex repeats and provide additional information on transcript isoforms [16, 26]. A limitation of the PacBio platform is its elevated sequence error rate; however, PacBio SMRT software can enhance sequencing data accuracy with the reads of insert (ROIs) algorithm, which generates a circular consensus sequence (CCS), thereby reducing sequencing errors and improving data quality [26, 27]. This sequencing technology is currently effective for full-length transcriptome profiling across various organisms [28, 29], such as plants (*Saccharum officinarum*), invertebrates (*Litopenaeus vannamei*), and vertebrates (*Misgurnus anguillicaudatus* and *Bungarus multicinctus*) [30–33]. Advances in single-molecule sequencing and functional analysis technologies have enabled a growing body of research into genome replication mechanisms [34, 35].

In this study, we generated the first full-length transcriptome of *I. braminus* using Iso-seq technology. The main goals were threefold: (1) establish a reference full-length transcriptome; (2) utilize this reference full-length transcriptome for phylogenetic analysis; and (3) explore a recent polyploidization event in *I. braminus*. Specifically, the aim of this study was to determine the timing of the genome duplication event. This study provides a comprehensive set of coding genes, offering preliminary evidence for the polyploidization of *I. braminus* at the molecular level. These data constitute an essential genetic resource that will facilitate further research in this field.

## Methods

### Sample collection and RNA extraction

Specimens were collected from Wenshan (Yunnan) and Wangmo (Guizhou) between 2019 and 2023. All individuals were maintained on moist soil in the laboratory before RNA extraction and chromosome preparation (animal handling and experiments were approved by the Ethics Committee at Guizhou Normal University, Permission number: 20,230,300,015). The experiments adhered to the Animal Research: Reporting of In Vivo Experiments (ARRIVE) guidelines [36]. All methods were conducted in compliance with relevant guidelines and regulations.

The specimens were euthanized using ethyl acetate and then preserved in 70% alcohol. For maximum mRNA extraction, five representative organs (brain, heart, liver, skin, and muscle) were collected and mixed from two healthy adult females. Total RNA was extracted from the mixed tissues using TRIzol reagent (Invitrogen, MA, USA) on dry ice, following the manufacturer’s instructions. DNA was removed using TURBO DNase I (Promega, Beijing, China). RNA degradation and contamination were assessed by 1% agarose gel electrophoresis. RNA purity was determined using a NanoDrop 2000 microspectrophotometer (Thermo Scientific, USA; NanoDrop 2000 detection blank reference: DEPC water). The RNA integrity (RIN) was accurately measured with an Agilent 4200 (Agilent Technologies). Only RNA samples with a RIN ≥ 8 were considered suitable for cDNA library construction.

### Library construction and PacBio sequencing

PolyA-containing mRNAs were enriched with oligo (dT) bead primers. The enriched mRNAs were reverse transcribed into cDNA using a Clontech SMARTer™ PCR cDNA Synthesis Kit (Clontech, CA, USA). Subsequently, the synthesized full-length cDNA was amplified via PCR. The cDNA fragments were purified using Pronex beads (Promega), with ratios varying according to transcript size. Purified cDNA was subjected to DNA damage repair, end repair, and ligation with SMRT dumbbell-type sequencing adapters. Following library construction, the Qubit 2.0 system (Life Technologies) was used for quantification, and the Agilent 2100 system (Agilent Technologies) was used to verify library insert size. The SMRTbell template was annealed with a sequencing primer, bound to polymerase, and sequenced using the PacBio Sequel II platform for data acquisition.

### Data processing and transcriptome assembly

High-quality CCSs were produced using the IsoSeq3 pipeline’s CCS command (https://github.com/PacificBiosciences/IsoSeq3), with the following parameters: min_predicted_accuracy 0.9 --min_passes 1 --top_passes 100 --min_length 200 --max_length 100,000. The construction of full-length transcripts involved four steps: (1) obtaining full-length reads by primer removal and demultiplexing using lima (v2.2.0, https://lima.how/); (2) classifying CCS reads into full-length nonchimeric (FLNC) and non-full-length (nFL) reads based on splice primer and chimaera presence; (3) further refining FLNC reads using IsoSeq3’s refine function (v3.4.0, parameter: --require_polya), involving polyA tail identification and removal; and (4) deriving the final full-length transcripts by clustering sequences with the IsoSeq3 clustering function (v3.4.0, parameter: --use_qvs). To ensure no possible contamination from other organisms, 1,000 random reads were aligned against the NCBI NT database using BLASTN [37] (e-value ≤ 1^e − 5^). The completeness of the transcriptome assembly was evaluated using the benchmarking universal single-copy orthologue (BUSCO, v5.2.2) [38].

### Gene annotation

Gene structures were annotated using a homologous protein-based method with Genewise (v2.4.1) [39] (parameter: --tfor_sum_genesf_Gff_subs 0.01 --indel 0.01 --trans_pseudo) and GeMoMa (v1.6.3) [40]. The reference protein sets used were obtained from *Python molurus bivittatus* (NCBI RefSeq GCF_000186305.1), *Deinagkistrodon acutus* (GigaDB, 10.5524/100196), and *Protobothrops mucrosquamatus* (NCBI RefSeq GCF_001527695.2). The gene structures from GeneWise and GeMoMa were merged and the longest protein at each locus was selected for final annotation. The remaining unannotated genes were further predicted de novo using TransDecoder.

Representative protein sequences were annotated using the following five functional databases. BLASTP (v2.7.1, e-value ≤ 1e^− 5^, identity ≥ 30% and subject coverage ≥ 30%) [37] was utilized to perform searches against the NCBI NonRedundant Protein (NR, http://www.ncbi.nlm.nih.gov) and SwissProt [41] protein databases. Kofam (v1.3.0, e-value ≤ 1e^− 5^) [42] for KO annotation in the Kyoto Encyclopedia of Genes and Genomes (KEGG) database [43]. Gene Ontology (GO) terms [44] and protein domain (ProDom) [45] were predicted by InterProScan (v5.2) [46].

### Identification of gene families

The protein sequences of 13 vertebrate species (including mammals, birds, amphibians, and reptiles) were downloaded from GigaDB or the NCBI (Supplementary Table S1). For gene loci with alternative splicing variants, only the longest transcript was selected. Genes with fewer than 50 amino acids were removed. Several closely related snake species and representative mammals, birds, amphibians, and reptiles were selected to ensure coverage of the major evolutionary clades. Self-to-self alignment of pooled protein sequences from species with available genomes was conducted using BLASTP (E-value of 1e^− 5^) [37], with low-quality hits being removed (identity < 30% and coverage < 30%) [47]. Orthologous groups were constructed from the filtered BLASTP results using OrthoFinder2 [48].

### Evolution analysis and divergence time estimation

Single-copy gene families were extracted from the OrthoFinder2 results for the 13 species. The protein sequences of *I. braminus* were aligned with those of the obtained single-copy gene families to extract the reciprocal best hits (RBHs). Subsequently, single-copy gene families for these 14 species were generated. Protein alignment for each single-copy family was conducted using MUSCLE (v3.8.31) [49]. The corresponding coding sequence (CDS) alignments were back-translated from the corresponding protein alignments using PAL2NAL [50]. Gblocks [51] was used to extract the conserved CDS alignments. For phylogenetic tree construction, a supermatrix was created by concatenating the CDS alignments of single-copy families. Maximum likelihood (ML) trees for supergenes constructed from full-length and 4DTv sites, were generated using the GTR + I + Γ model with RaxML [52]. The three codon positions in the concatenated supermatrix were treated as separate partitions due to significant differences in evolutionary rates, corresponding to the 1st, 2nd and 3rd codon sites of the CDS. Divergence dates were estimated using a relaxed clock model via the PAML4.7 package [53]. The “independent rates model (clock = 2)” and “JC69” models in the MCMCTREE program were used, running six million MCMC iterations after two million burn-in iterations [47]. For consistency, each data type was run twice through the program. In the first run, the chronogram was generated using FigTree (v1.4.0, http://tree.bio.ed.ac.uk/).

### Polyploidy analysis

Gene families encompassing 14 species were constructed using OrthoFinder2. In each family, all paralogues from *I. braminus* were retained, whileonly the longest paralogue was retained for the other 13 species. Proteins in each family were aligned with MUSCLE (v3.8.31) [49] using default parameters, and CDS alignments were generated from these using PAL2NAL [50]. The ML phylogenetic tree was constructed using RAxML [52] with the GTR + I + Γ model. Gene trees that conflicted with the species tree were filtered out. The divergence times in the gene tree were estimated using the MCMCTREE program in the PAML4.7 package [53]. MCMCTREE operated similarly to the above description, with the exception that CDS alignments were not partitioned [47].

The synonymous mutation rate (*Ks*) distribution of paralogues is commonly used to infer whole-genome duplications (WGDs) [54, 55]. *Ks* distributions for *I. braminus*, *P. bivittatus*, *Xenopus laevis* (2*n* = 4x = 38), and *Xenopus tropicalis* (2*n* = 2x = 18) were obtained using the WGDdetector [55]. The WGDdetector pipeline integrates gene family construction and *Ks* value estimation for paralogues pairs, plotting the distribution using an R script [55]. *Ks* plots indicate of past duplications, while karyotyping analysis directly infers contemporary polyploidy. Chromosomes were prepared according to a standardized procedure [56]. For this experiment, a healthy adult female snake underwent an intraperitoneal injection of 0.1% colchicine for 8 h. After the snakes were euthanized with ethyl ether vapour, the digestive tract was dissected and immersed in 0.6% normal saline (NS). Intestinal samples were sectioned into small pieces and treated with hypotonic KCl (0.075 M). The cells were fixed in fresh cool Carnoy’s fixative (glacial acetic acid/methanol, 1:3). Chromosome suspensions were prepared by dropping them onto clean slides, followed by staining and banding after drying. Conventional staining was achieved using 20% Giemsa solution for 3–5 min. After staining, the slides were rinsed thoroughly with running water and dried before imaging or observation. Twenty optimal metaphase plates were selected for photographic analysis using a 100x objective microscope (E100, Nikon). Chromosomes were classified according to Levan et al. [57], and karyotype measurements were conducted using ImageJ software [58].

## Results

### Data summary

**Fig. 1:**
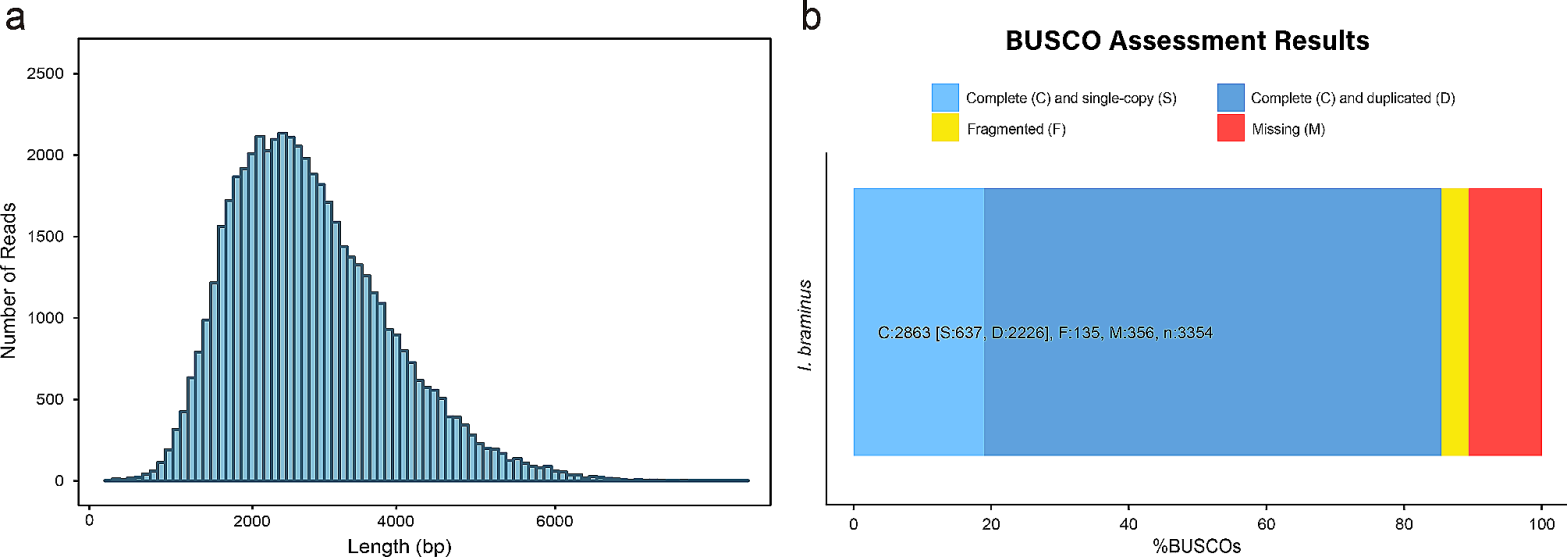
Length distribution (**a**) and integrity assessment (**b**) of *I. braminus* transcripts

A total of 96 Gb of raw sequencing data was obtained from Iso-seq using the PacBio SMRT sequencing method. Following initial quality control, which involved the removal of adapter sequences and subreads < 50 bp in length, a total of 43,131,390 subreads with an average length of 2,226 bp were generated (Table 1 and Supplementary Fig. S1a). To assess accuracy, 1,000 random sequences were aligned to the NCBI NT database; 98% of the sequences were identified, most of which were similar to closely related reptiles. Thereafter, all subreads underwent CCS analysis, which produced 1,356,270 CCSs averaging 2,513 bp in length (Table 1 and Supplementary Fig. S1b). The FLNC reads were clustered usingthe cluster function of IsoSeq3 to correct errors in the third-generation sequencing data. Ultimately, 51,849 full-length transcriptomes were obtained, averaging 2,719 bp in length, with a maximum of 9,319 bp, an N90 of 1,791 bp, and an N50 of 2,980 bp (Fig. 1a). Thecompleteness of the assembly was 89.4% as determined by BUSCO, with 19% as complete and single-copy BUSCOs, 66.4% as complete and duplicated BUSCOs, and 4% as fragmental BUSCOs (Fig. 1b).

**Table 1:** Assembly statistics for *I. braminus* transcriptome

**Subreads**	
Number of reads	43,131,390
Number of bases sequenced (bp)	96,030,005,322
Average length (bp)	2,226
**CCS**	
Number of reads	1,356,270
Number of CCS bases (bp)	3,407,675,187
Average length (bp)	2,513
**Full-length transcriptome**	
Total number	5,1849
Total length (bp)	141,014,862
Average length (bp)	2,719
Max length (bp)	9,319
Min length (bp)	87
N50 length (bp)	2,980
N90 length (bp)	1,791
GC content (%)	47.9

### Coding sequence prediction and gene annotation

**Fig. 2:**
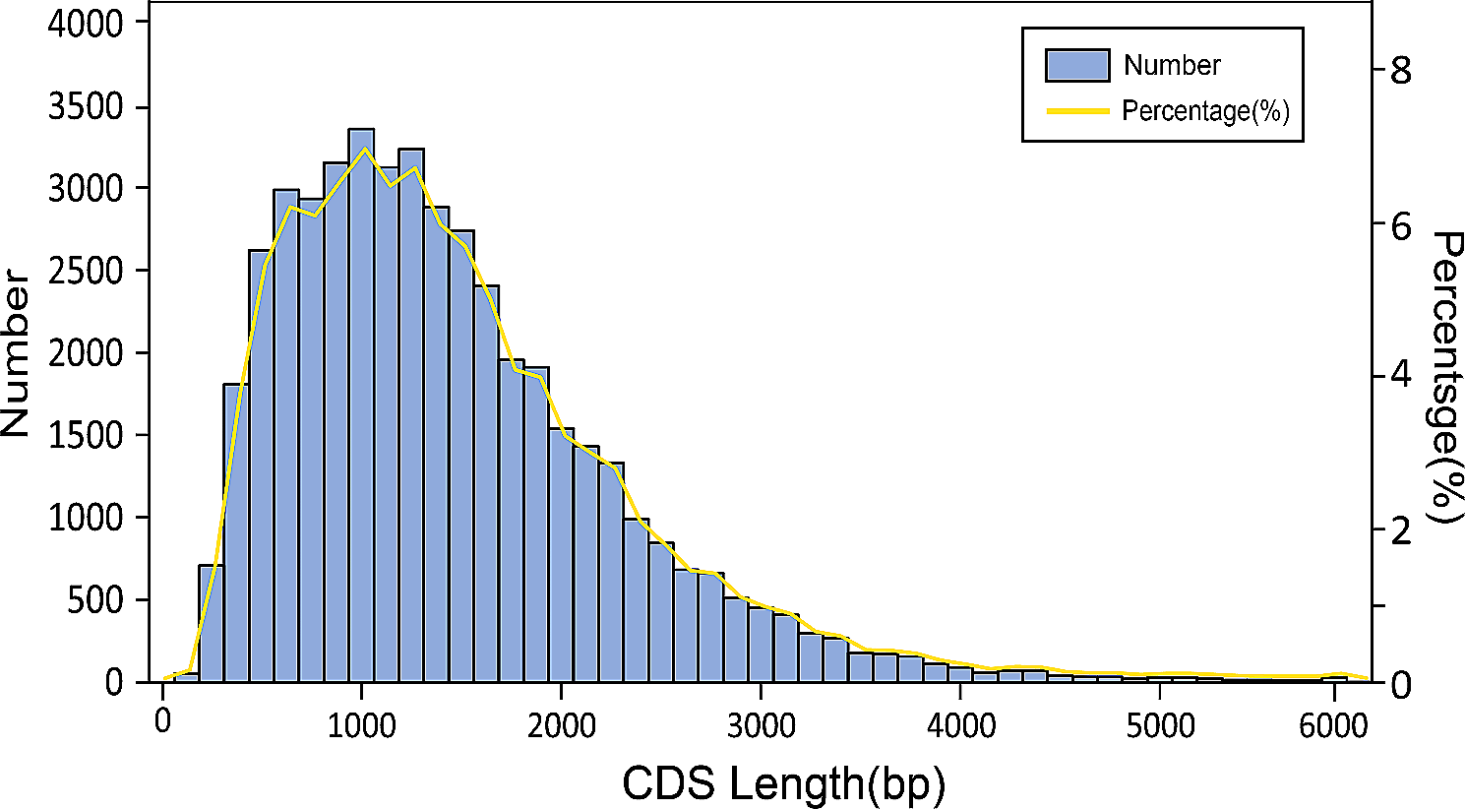
Number, percentage and length distributions of coding sequences of *I. braminus* transcripts

Identifying CDSs is crucial for gene annotation, aiding preliminary gene structure analysis and providing valuable insights for functional annotation and evolutionary analysis [59, 60]. In this study, the full-length transcripts were annotated based on protein sequence information from homologous species. A total of 46,660 (89.99%) transcripts were successfully annotated, via the use of three software tools. The CDS lengths ranged from 36 to 7,875 bp, with an average length of 1,383 bp (Fig. 2). Among these sequences, 35,481 (68.43%) CDS transcripts matched the reference protein sequence, and 34,633 (66.8%) not only aligned with the reference but also had only one terminator.

**Fig. 3:**
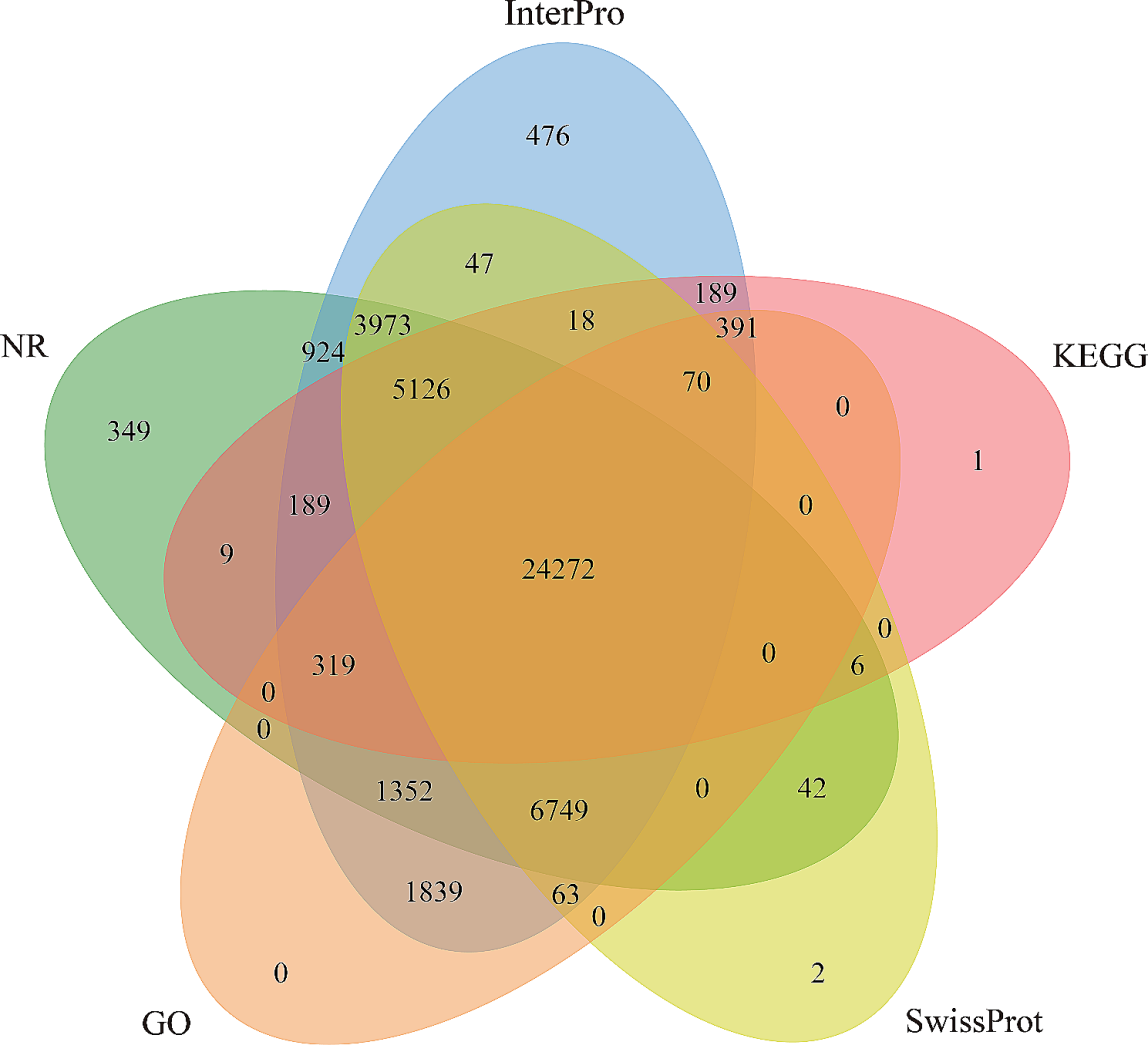
Venn diagram of the annotations between the InterPro, NR, GO, KEGG, and Swiss-Prot databases

**Fig. 4:**
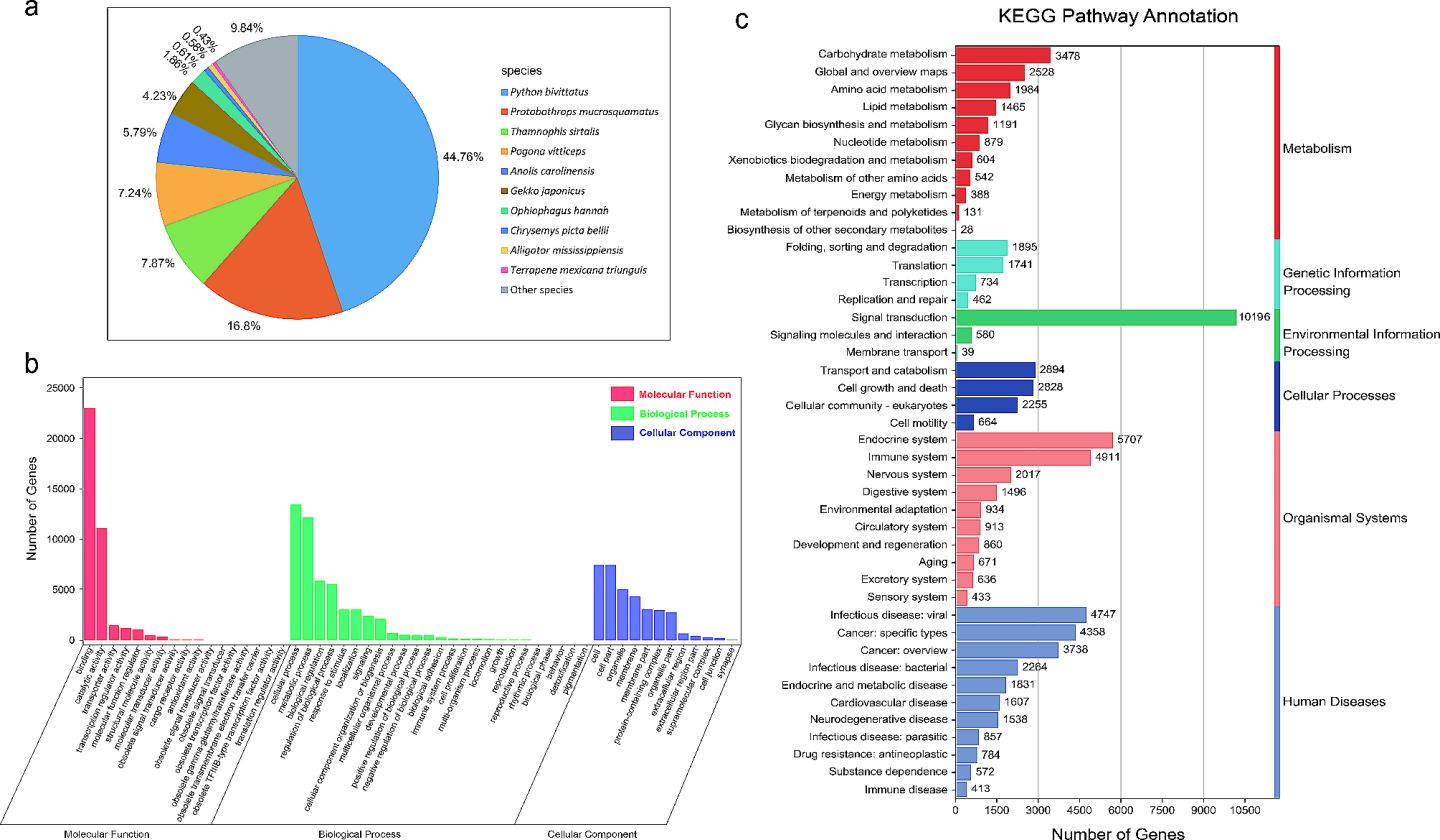
Gene functional annotations in the public databases. **a** Distribution of homologous species annotated in the NR database (the first three species belonging to snakes); **b** Distribution of functional classifications based on GO terms; **c** Distribution of pathway classifications based on the KEGG database

Full-length nonredundant transcripts were annotated using five databases, and 46,406 (99.46%) transcripts were successfully identified. Functional annotation revealed 45,997 (98.58%), 43,368 (92.82%), 40,368 (86.52%), 35,055 (75.12%), and 30,590 (65.56%) transcripts annotated in the Iprscan, Nr, Swiss-Prot, GO, and KEGG databases, respectively (Fig. 3). According to the NR database, most transcripts were annotated to the Pythonidae and Viperidae families. The 5 most common annotated species were *P. bivittatus* (19,383, 44.76%), *Protobothrops mucrosquamatus* (7,274, 16.8%), *Thamnophis sirtalis* (3,409, 7.87%), *Pogona vitticeps* (3,136, 7.24%), and *Anolis carolinensis* (2,509, 5.79%) (Fig. 4a). With regard to GO annotation, the most enriched terms in the biological processes category were cellular process (13,498, 26.35%) and metabolic process (12,176, 23.77%). Within the molecular function category, the most enriched GO terms were binding (23,037, 58.83%) and catalytic activity (11,110, 28.37%). In the cellular component category, the most abundant GO terms were cell (7,456, 21.39%) and cell part (7,456, 21.39%) (Fig. 4b). According to the KEGG pathway annotation, 30,590 isoforms were annotated and assigned to 43 biological pathways (Fig. 4c). Numerous annotated isoforms were classified into pathways related to environmental information processing, organismal systems, and metabolism processing. In particular, 10,196 (12.94%) isoforms were associated with “signal transduction”, indicating the importance of signal transduction-related genes in *I. braminus*.

### Gene duplication

**Fig. 5:**
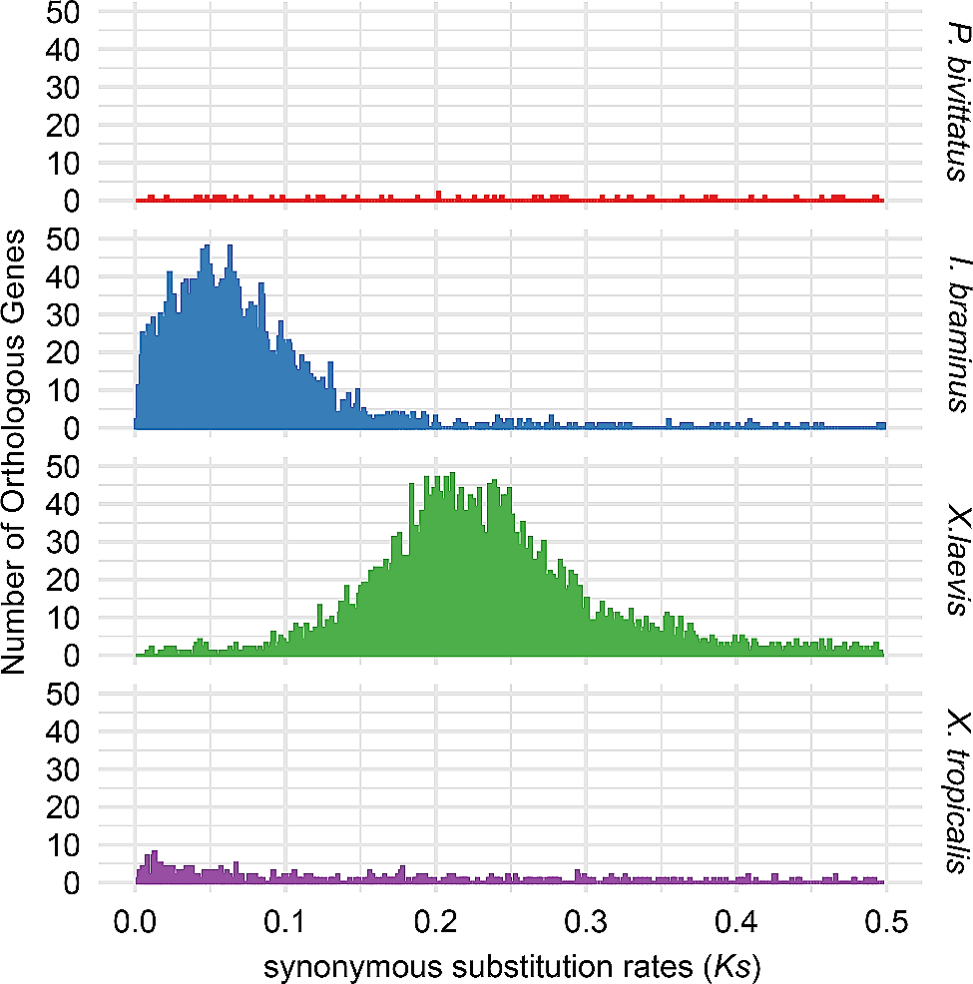
Distributions of synonymous substitution rates (*Ks*) between paralogous genes of *P. bivittatus*, *I. braminus*, *X. laevis*, and *X. tropicalis*

A large proportion of duplicate BUSCOs were identified in the integrity assessment of the full-length transcriptome. Of the 2,998 (89.4%) BUSCO groups in the transcriptome, 2,226 (66.4%) were duplicated, indicating that the gene duplication possibly resulted from WGD (Fig. 1b). We used WGDdetector [55] to estimate *Ks* values for four species (*I. braminus*, *P. bivittatus*, *X. laevis* (tetraploid), and *X. tropicalis*), and their *Ks* distributions were plotted. The *Ks* plots showed a clear *Ks* peak for *X. laevis* and *I. braminus* (Fig. 5). This finding suggested a recent gene duplication event in *I. braminus*.

### Karyological analysis

**Fig. 6:**
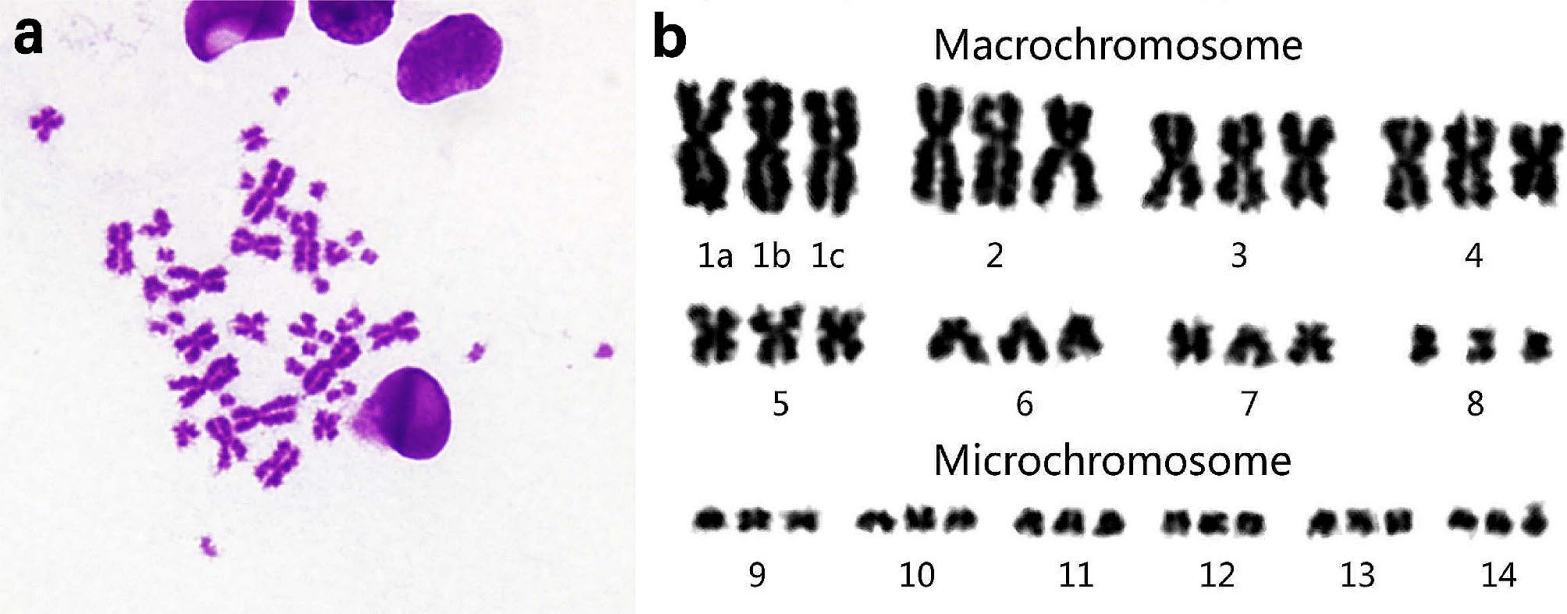
Metaphase chromosome plates (**a**) and standardized karyotypes (**b**) of *I. braminus* according to conventional staining

The examined female specimens of *I. braminus* had karyotypes of 3*n* = 42 chromosomes, with 8 macrochromosome triplets and 6 microchromosome triplets. This alignment was consistent with findings from previous studies by Ota et al. [3] and Patawang et al. [61]. Among the macrochromosomes, the first four pairs were larger and metacentric, while the other four included two metacentric pairs (pairs 5 and 8) and two submeta-subtelocentric pairs (pairs 6 and 7), as shown in Fig. 6. The fundamental number (NF, number of chromosome arms) was 60, and the karyotypic formula was as follows: 3*n* (42) = L^m^_12_+S^m^_6_+S^sm^_4_+S^st^_2_+18 microchromosomes.

### Phylogenetic analyses and divergence time estimation

**Fig. 7:**
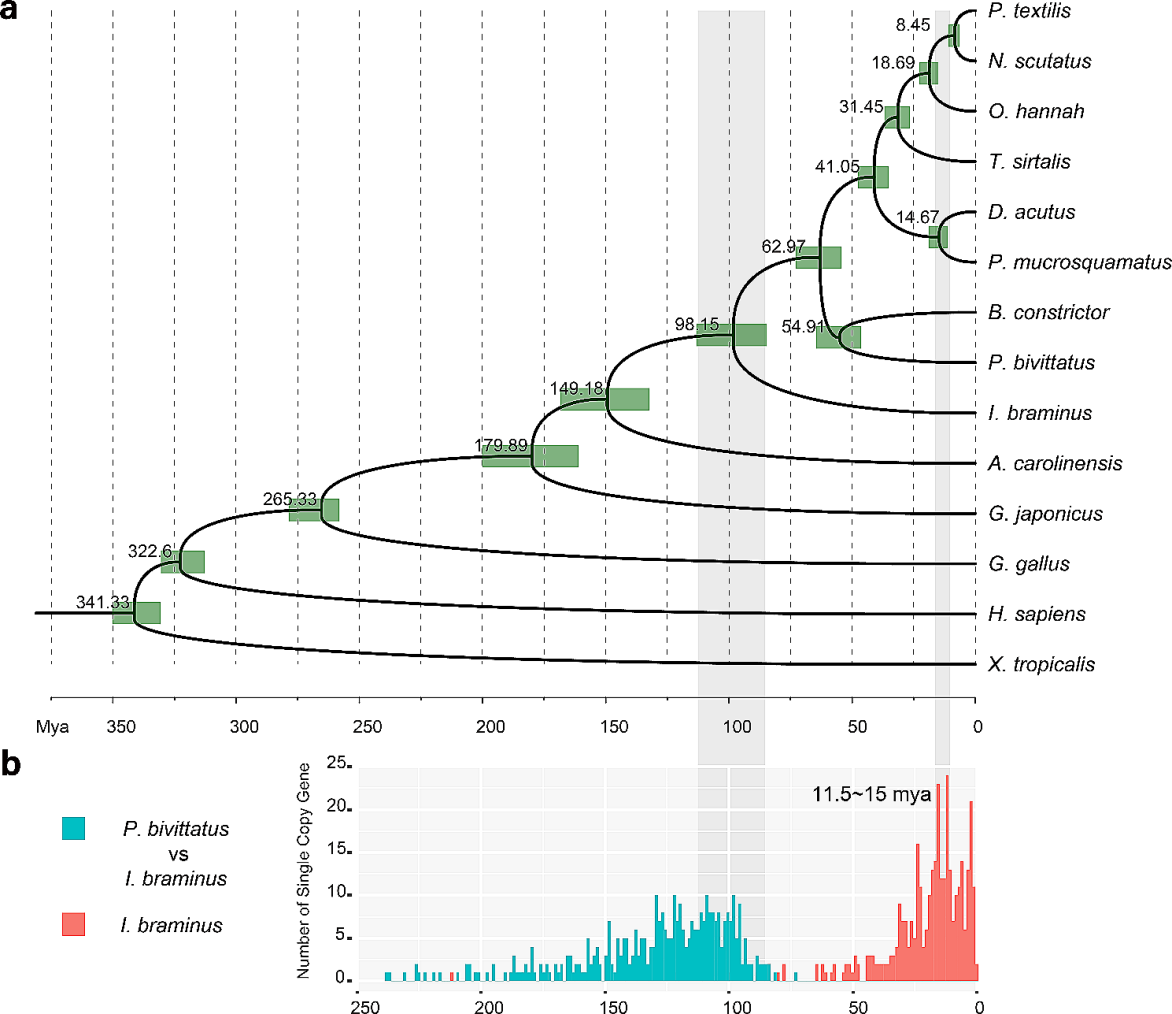
Phylogenetic tree and divergence dates of 14 species. **a** Phylogenetic tree highlighting the phylogenetic position of *I. braminus*. The green bars are the time ranges of the divergence dates. The grey boxes correspond to the divergence date of *I. braminus-P. bivittatus*; **b** Divergence date of *I. braminus-P. bivittatus* (blue); The estimated divergence dates of *I. braminus* subgenomes (red). The total number of single-copy genes for *I. braminus-P. bivittatus* was 1826

The OrthoFinder2 results revealed 3,249 single-copy gene families across 13 species (Supplementary Table S2). The protein sequences of *I. braminus* were aligned with those of single-copy gene families to extract RBHs, identifying 1,826 single-copy gene families across 14 species. Phylogenetic trees were constructed using the ML method in RaxML. The phylogenetic tree aligns with the snake suborder estimates provided by Yan et al. [62] and Liu [63] based on mitochondrial genomes (Fig. 7a). The bootstrap support value for each branch was 100 (Supplementary Fig. S2). The phylogenetic tree showed that nine snake species formed a monophyletic clade, with *I. braminus* diverging the earliest. The position of *I. braminus*, as the sister lineage to the other eight snakes, suggested its more ancient position in the evolutionary history of snakes.

Divergence dates were estimated under a relaxed clock model using the MCMCTREE program in the PAML4.7 package. Time calibration of the estimated tree was also conducted (Supplementary Table S3). Divergence dates for the 14 species were determined to be within a certain range (noted by green bars in Fig. 7a). In each gene family, all *I. braminus* paralogues were retained, while only the longest paralogue was retained in the other 13 species. The gene divergence time was estimated according to previous methods, except for CDS alignment partitioning, which was not performed. The results revealed that the divergence between *I. braminus* and *P. bivittatus* occurred ~ 98.15 Mya (Fig. 7a), and the divergence of *I. braminus* subgenomes occurred more recently, approximately 11.5 ~ 15 Mya (Fig. 7b).

## Discussion

Transcriptome analysis provides crucial insights into genomic characteristics, including genome duplication [64]. Iso-seq, a third-generation sequencing technology, has emerged as a potent tool in transcriptomics due to its single-molecule sequencing and long read capabilities [65]. In recent years, Iso-seq has greatly enhanced our understanding of the complex nature of the transcriptome. In this study, the Iso-seq platform was used to sequence and analyse the full-length transcriptome of *I. braminus*. Through assembly and splicing, 51,849 transcripts were ultimately obtained. The majority of the reads exhibited high accuracy, with most having a Phred quality score above 20 (indicating an error probability of 1%) and some above 60 (indicating an even lower error probability), emphasizing the reliability of the full-length transcript data. Subsequent transcript annotation, via multiple databases, provided deeper insights into the structure and function of the transcripts. According to the NR database, the species most closely related to *I. braminus* was *P. bivittatus.* Among the full-length transcriptome of *I. braminus*, 27,707 transcripts were annotated in the GO database; these genes were associated predominantly with biological processes, followed by molecular functions and cellular components. A total of 47,197 *I. braminus* transcripts were annotated in 43 KEGG pathways, with the top four pathways being involved in signal transduction, the endocrine system, the immune system, and infectious disease (viral). These findings highlight the importance of our PacBio transcript data as valuable resources and references for future studies, particularly in annotating reptile gene structures, conducting functional analysis, and performing pathway research. Additionally, our results enrich the genetic knowledge of *I. braminus*, aiding future research into genes related to snake development, reproduction, and evolution.

Polyploidization, or WGD, is a typical feature of eukaryotic evolution, thought to confer selective benefits to polyploids and play a key role in speciation and eukaryotic development [66, 67]. For instance, Wang et al. [68] showed that, compared with *Danio rerio*, *Cyprinus carpio* has experienced an additional WGD event, resulting in the divergence of common carp as an independent species from its common ancestor. Multiple genome duplication events occurred during the evolution of chordates, with some occurring near the origin of vertebrates [69, 70]. Our study revealed the presence of many duplicated genes in the *I. braminus* transcriptome, based on BUSCO assessments (Fig. 1b) and the *Ks* peak detected by the WGDdetector (Fig. 5). Furthermore, the gene count in *I. braminus* (46,660) surpassed that of other snakes, possibly due to genome duplication. Dating analysis using MCMCTREE suggested at least two distinct subgenomes in *I. braminus* (Fig. 7b). Integrating these findings with the karyotyping results, it can be concluded that *I. braminus* is triploid. *Ks* plots provide evidence of past duplication, while karyotyping results help identify contemporary polyploidy. Although each of these approaches has limitations, we considered putative polyploidization to be supported when these results were consistent. The most reliable evidence for WGD requires synteny-based analysis with high-quality whole genomes (three haplotypes) [71]. Therefore, further genomic studies are necessary to fully elucidate the mechanisms driving these gene duplication events.

Polyploidy occurs more frequently in plants than in animals. It is observed in only a few species of insects, bony fish, amphibians, and reptiles [66, 67]. The reason for the scarcity of animal polyploidy was first proposed by Muller [72], who proposed that changes in chromosomes may impact reproductive mechanisms or sex determination. Consequently, polyploidy is generally perceived as an evolutionary blind alley, primarily due to its association with unisexual reproduction [73, 74]. However, extensive research on polyploidy has shown that many animals exist as stable polyploids [66, 75]. Polyploids such as *I. braminus* thrived in terms of survival and reproduction, and even this unisexual polyploid species has existed for millions of years. In the book “*The Evolution of the Genome*,” Gregory et al. [76] emphasized the potential advantages of polyploidy, including increased adaptability to harsh conditions and wider geographic ranges, increasing resistance to extinction and facilitating genealogical selection. The divergence of *I. braminus* subgenomes took place during the middle Miocene (11 ~ 17 Mya), coinciding with significant climatic events such as the Miocene Climatic Optimum (MCO) and a subsequent sudden cooling and Antarctic ice-sheet expansion phase, called the Middle Miocene Climate Transition (MMCT) [77, 78]. Global climatic changes during the Miocene period are hypothesized to have influenced the evolutionary trajectory of *I. braminus*, but the precise mechanisms underlying this phenomenon remain to be elucidated. Fundamental questions about *I. braminus* persist, including the mechanism of polyploidization, the consequences of genome duplication, and gene interactions. Thus, there is a notable research gap regarding the genetic aspects of *I. braminus* polyploidy; revealing its evolutionary history and genomic characteristics in the postgenomic era is urgent. Notably, the recent publication of the draft genome sequence of *I. braminus* in Scientific Data offers a valuable resource for future research endeavours. Future efforts will focus on mapping transcripts to this genome assembly and establishing gene models related to the draft genome. In the context of rapid growth in molecular technology, using the transcriptome or genome sequence as an entry point for analysis may lead to additional insights.

## Conclusions

In this study, we successfully obtained the full-length transcriptome of *I. braminus* using Iso-seq high-throughput sequencing technology, thereby providing a novel perspective for confirming the polyploidization of *I. braminus*. Our analysis provides preliminary evidence supporting a genome duplication event in *I. braminus*, with an estimated divergence date of its subgenomes between 11.5 and 15 Mya. These results provide valuable insights for future research into snake transcriptomes and genomes, aiding the exploration of other polyploid vertebrates. Additionally, this study has the potential to broaden the application of PacBio sequencing in vertebrate transcriptome research.

### Electronic supplementary material

Below is the link to the electronic supplementary material.

Supplementary Material 1

## Data Availability

The transcript sequences from the PacBio Iso-seq transcriptome are available at the NCBI Sequence Read Archive (SRA accession SRR24061511 https://www.ncbi.nlm.nih.gov/sra/SRR24061511).
