# Supplementary material for: Polyploidization of Indotyphlops braminus: evidence from isoform-sequencing
Source: BMC Genom Data. 2024 Feb 26;25:23. doi: 10.1186/s12863-024-01208-y (PMC10895795; doi:10.1186/s12863-024-01208-y)
Supplement: Supplementary file 1 — Supplementary Material 1 [file 12863_2024_1208_MOESM1_ESM.docx]

Polyploidization of *Indotyphlops braminus*, evidence from Isoform-sequencing

Supplementary information

**contents**

**Table S12**

**Table S22**

**Table S33**

**Figure S14**

**Figure S24**

**Table S1** Information of the species used for the evolution analysis.

| Latin name | Latin name (abbreviation) | Data source |
| --- | --- | --- |
| *Anolis carolinensis* | *A. carolinensis* | GCF_000090745.1 (NCBI RefSeq) |
| *Boa constrictor* | *B. constrictor* | <http://dx.doi.org/10.5524/100196> (GigaDB) |
| *Deinagkistrodon acutus* | *D. acutus* | <http://dx.doi.org/10.5524/100196> (GigaDB) |
| *Gallus gallus* | *G. gallus* | GCF_016699485.2 (NCBI RefSeq) |
| *Gekko japonicus* | *G. japonicus* | GCF_001447785.1 (NCBI RefSeq) |
| *Homo sapiens* | *H. sapiens* | GCF_000001405.40 (NCBI RefSeq) |
| *Notechis scutatus* | *N. scutatus* | GCF_900518725.1 (NCBI RefSeq) |
| *Ophiophagus hannah* | *O. hannah* | GCA_000516915.1 (NCBI GenBank) |
| *Python bivittatus* | *P. bivittatus* | GCF_000186305.1 (NCBI RefSeq) |
| *Protobothrops mucrosquamatus* | *P. mucrosquamatus* | GCF_001527695.2 (NCBI RefSeq) |
| *Pseudonaja textilis* | *P. textilis* | GCF_900518735.1 (NCBI RefSeq) |
| *Thamnophis sirtalis* | *T. sirtalis* | GCF_001077635.1 (NCBI RefSeq) |
| *Xenopus tropicalis* | *X. tropicalis* | GCF_000004195.4 (NCBI RefSeq) |
| *Indotyphlops braminus* | *I. braminus* | this paper |

**Table S2** Statistics of the gene families.

| Species | #Genes | #Unclustered | #Genes in families | #Family | #Unique  genes | Common | | #Single  copy |
| --- | --- | --- | --- | --- | --- | --- | --- | --- |
|  |  |  |  |  |  | #Family | #Genes |  |
| *A. carolinensis* | 19,100 | 724 | 18,376 | 13,618 | 71 | 7,196 | 10,523 | 3,249 |
| *B. constrictor* | 18,957 | 935 | 18,022 | 13,788 | 41 | 7,196 | 10,603 | 3,249 |
| *D. acutus* | 20,019 | 554 | 19,465 | 13,391 | 40 | 7,196 | 10,680 | 3,249 |
| *G. gallus* | 16,852 | 1,037 | 15,815 | 12,311 | 284 | 7,196 | 9,808 | 3,249 |
| *G. japonicus* | 19,374 | 432 | 18,942 | 13,667 | 24 | 7,196 | 11,160 | 3,249 |
| *H. sapiens* | 22,386 | 2,221 | 20,165 | 13,362 | 208 | 7,196 | 11,861 | 3,249 |
| *N. scutatus* | 19,449 | 352 | 19,097 | 13,941 | 26 | 7,196 | 11,014 | 3,249 |
| *O. hannah* | 18,388 | 3,250 | 15,138 | 11,597 | 65 | 7,196 | 9,697 | 3,249 |
| *P. bivittatus* | 19,672 | 384 | 19,288 | 14,267 | 18 | 7,196 | 11,299 | 3,249 |
| *P. mucrosquamatus* | 19,933 | 461 | 19,472 | 13,514 | 28 | 7,196 | 11,682 | 3,249 |
| *P. textilis* | 19,007 | 177 | 18,830 | 13,894 | 22 | 7,196 | 10,804 | 3,249 |
| *T. sirtalis* | 18,276 | 754 | 17,522 | 13,152 | 31 | 7,196 | 10,609 | 3,249 |
| *X. tropicalis* | 20,939 | 1,858 | 19,081 | 13,084 | 202 | 7,196 | 11,239 | 3,249 |

^#The “Genes” subtext indicates that the column includes the number of genes in a gene family, and the “Family” subtext indicates that the column includes the number of gene families. “Unclustered” indicates genes that could not be clustered into any gene family. “Unique” indicates a gene family for which only one species exists. “Common” indicates a gene family in which all the species are present, and “Single copy” indicates a gene family in which only one gene exists.^

**Table S3** Calibrating points for estimating divergence times

| Clade | Clade | Min  (100 Myr) | Max  (100 Myr) | Reference |
| --- | --- | --- | --- | --- |
| *Homo sapiens* | *Xenopus tropicalis* | 3.304 | 3.501 | [1]^[[1]](#footnote-1)^ |
| *Homo sapiens* | *Gallus gallus* | 3.123 | 3.304 | [1] |
| *Gallus gallus* | *Anolis carolinensis* | 2.597 | 2.998 | Timetree |

^Myr, million years.^

**
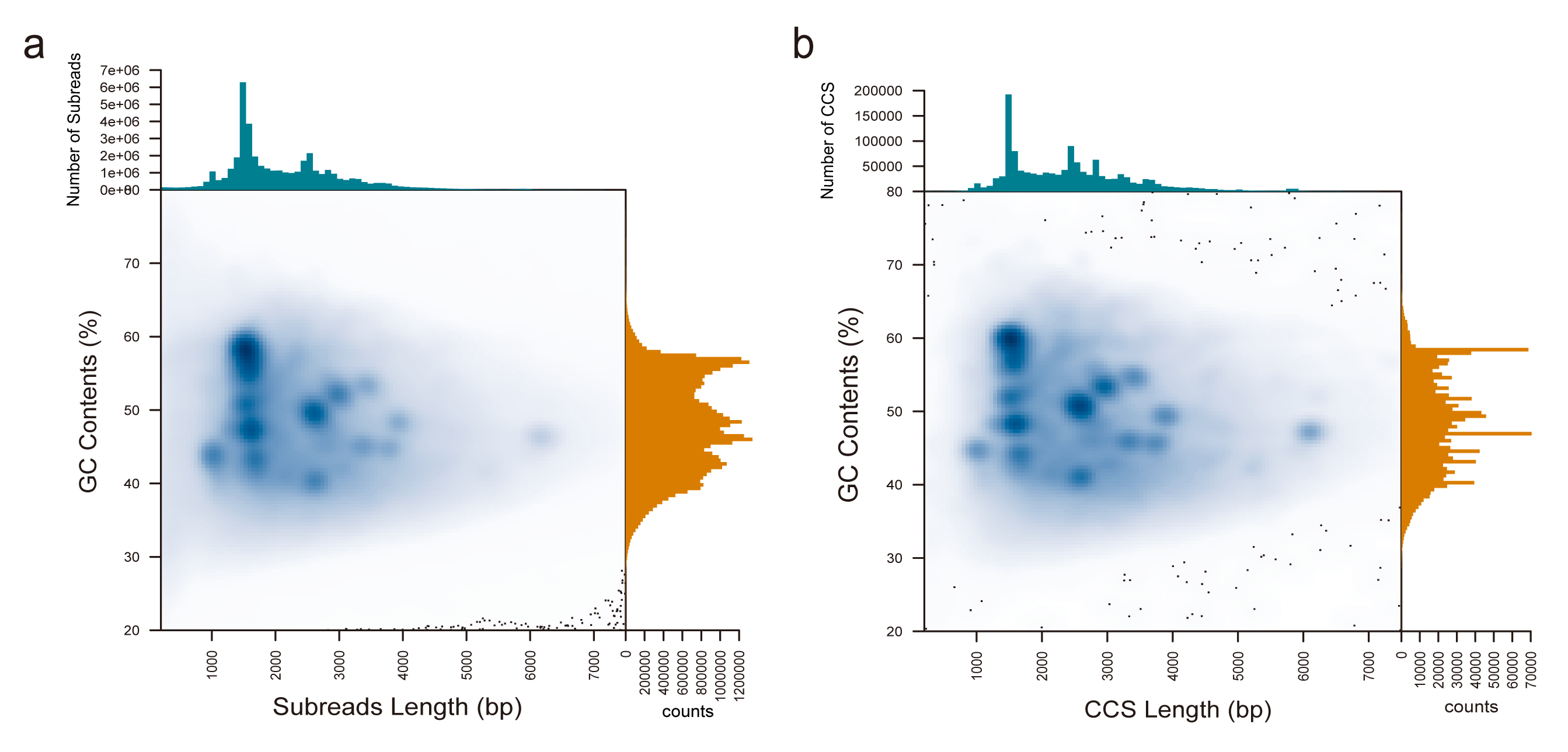
F****ig. S1** **a** Length distribution and GC contents of subreads; **b** Length distribution and GC contents of CCS reads

**
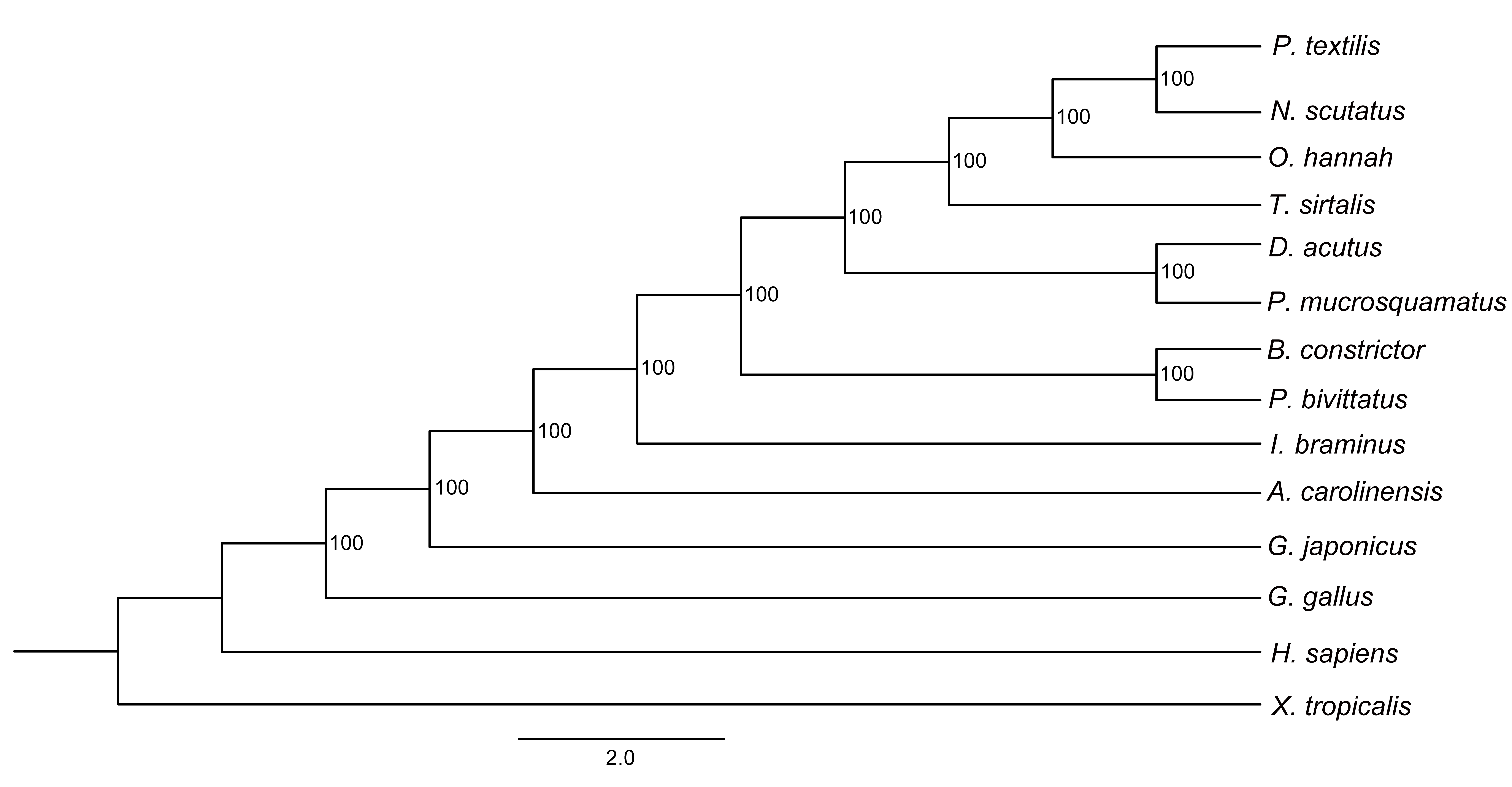
**

**Fig. S2** Maximum likelihood trees were constructed using RaxML using the 4DTv sites. The bootstrap values are labeled on each node

1. 1. Benton MJ, Donoghue PC. Paleontological evidence to date the tree of life. Mol Biol Evol. 2007;24:26-53. [↑](#footnote-ref-1)
